# Two Novel Mutations of *ANKRD11* Gene and Wide Clinical Spectrum in KBG Syndrome: Case Reports and Literature Review

**DOI:** 10.3389/fgene.2020.579805

**Published:** 2020-11-11

**Authors:** Su Jin Kim, Aram Yang, Ji Sun Park, Dae Gyu Kwon, Jeong-Seop Lee, Young Se Kwon, Ji Eun Lee

**Affiliations:** ^1^Department of Pediatrics, Inha University Hospital, Inha University College of Medicine, Incheon, South Korea; ^2^Department of Pediatrics, Kangbuk Samsung Hospital, Sungkyunkwan University School of Medicine, Seoul, South Korea; ^3^Department of Orthopaedic Surgery, Inha University Hospital, Inha University College of Medicine, Incheon, South Korea; ^4^Department of Psychiatry, Inha University Hospital, Inha University College of Medicine, Incheon, South Korea

**Keywords:** ANKRD11 gene, KBG syndrome, phenotype (mesh), growth hormone, intellectual disability—genetics

## Abstract

**Background:**

KBG syndrome (OMIM #148050) is a rare, autosomal dominant inherited genetic disorder caused by heterozygous mutations in the ankyrin repeat domain-containing protein 11 (ANKRD11) gene or by microdeletion of chromosome 16q24.3. It is characterized by macrodontia of the upper central incisors, distinctive facial dysmorphism, short stature, vertebral abnormalities, hand anomaly including clinodactyly, and various degrees of developmental delay. KBG syndrome presents with variable clinical feature and severity among individuals. Here, we report two KBG patients who have different novel heterozygous mutations of ANKRD11 gene with wide range of clinical manifestations.

**Case presentation:**

Two novel heterozygous mutations of ANKRD11 gene were identified in two unrelated Korean patients with variable clinical presentations. The first patient presented with short stature and early puberty and was treated with growth hormone and gonadotropin-releasing hormone agonist without adverse effects. He had mild intellectual disability. In targeted exome sequencing, a novel *de novo* frameshift variant was identified in ANKRD11, c.5889del, and p. (Ile1963MetfsX9). The second patient had severe intellectual disability with epilepsy. He had normal height and prepubertal stage at the age of 11 years. He had behavioral problems such as autism-like features, anxiety, and stereotypical movements. Whole exome sequencing (WES) was performed, and the novel heterozygous mutation, c3310dup, p. (Glu110GlyfsTer5) in ANKRD11 was identified.

**Conclusion:**

KBG syndrome is often underdiagnosed because of its non-specific features and phenotypic variability. Performing a next—generation sequencing panel, including the ANKRD11 gene for cases of developmental delay with/without short stature may be helpful to identify hitherto undiagnosed KBG syndrome patients.

## Introduction

KBG syndrome (OMIM #148050) is a rare, autosomal dominant inherited genetic disorder that is caused by heterozygous mutations in the ankyrin repeat domain-containing protein 11 (ANKRD11) gene (Sirmaci et al., 2011) or by microdeletion of chromosome 16q24.3 that includes the ANKRD11 gene (Willemsen et al., 2010; Isrie et al., 2012). The syndrome is characterized by macrodontia of the upper central incisors, distinctive facial dysmorphism (including triangular face, brachycephaly, synophrys, and hypertelorism), skeletal abnormalities (costovertebral anomalies, brachydactyly, scoliosis, short stature), developmental delays, and behavioral problems (Brancati et al., 2006; Morel Swols et al., 2017). In 2011, Sirmaci et al. demonstrated that mutations in the ANKRD11 gene caused KBG syndrome. To date, more than 200 patients with KBG syndrome have been reported; this has enabled clinicians to update the clinical features and genetic aspects of the condition (Scarano et al., 2019). Patients with KBG syndrome have diversiform phenotypes and various degrees of intellectual disability (ID), even within families where multiple members are affected (Morel Swols and Tekin, 2018). The Deciphering Developmental Disorders (DDD) study in the United Kingdom reported that about 1% of patients with an undiagnosed neurodevelopmental disorder were found to have mutations in the ANKRD11 gene (Wright et al., 2015). KBG syndrome is still likely to be underdiagnosed because of its various and non-specific symptoms, phenotypic overlap with other syndromes, and often mild clinical manifestations. Here, we report two cases of KBG syndrome; both were identified with different novel heterozygous variants of the ANKRD11 gene and showed a broad range of clinical manifestations.

## Case Presentations

### Patient 1

The patient was born at term via a cesarean section to healthy and non-consanguineous Korean parents, with a birth weight of 2,520 g (−1.76 standard deviation score, SDS) and without any perinatal problems. He had a medical history of recurrent otitis media and had undergone surgery to insert tympanostomy tubes in both ears. At the age of 5.5 years, he had a short stature and his bone age was more than 3.5 years behind the chronological age (according to the Greulich and Pyle atlas). At that time, he had been diagnosed with growth hormone (GH) deficiency (peak GH levels from two different GH stimulation test results were 7.71 and 4.65 ng/mL) and had been treated with GH. He showed a significant increase in growth velocity and height SDS (−3.04 to −1.91 SDS) after 3 years of GH treatment.

**FIGURE 1:**
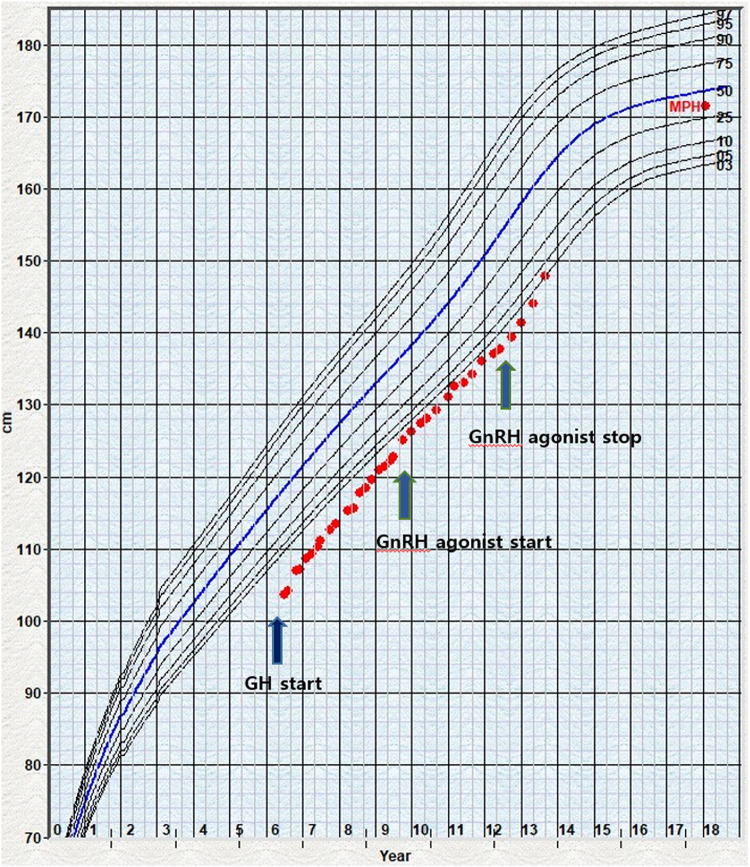
Growth chart of the patient 1 under GH and GnRH agonist treatment. The dot indicates the height measurement. The arrow marks the beginning of GH and GnRH treatment start or stop. GH, growth hormone; GnRH, gonadotropin-releasing hormone; MPH, mid-parental height.

**FIGURE 2:**
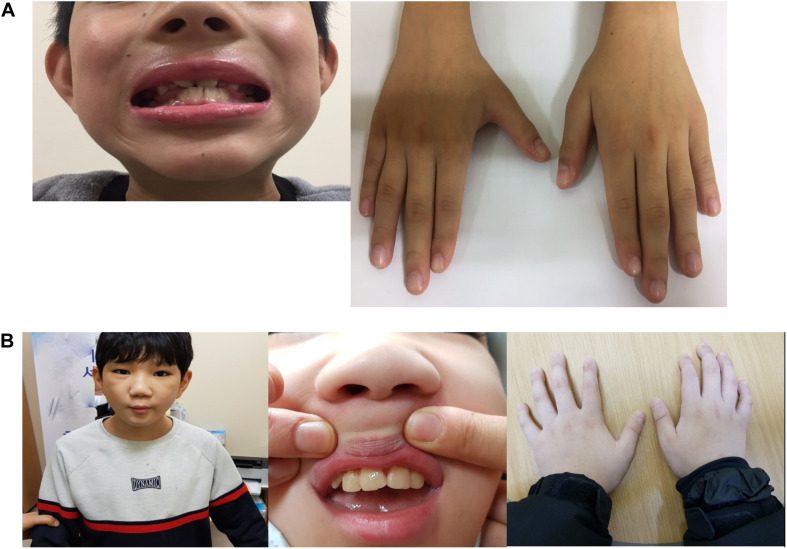
**(A)** Dysmorphic features of patient 1. Macrodontia of the upper central incisors, a dysmorphic face including prominent and high nasal bridge, anteverted nostrils, hypertelorism, long philtrum, and brachydactyly of both hands. **(B)** Dysmorphic features of patient 2. Macrodontia of the permanent upper central incisors; dysmorphic face features such as triangular face, thick eye lashes and eyebrows, prominent nasal bridge, long philtrum, and brachycephaly; and hand anomalies including brachydactyly and clinodactyly of the 5th finger.

At the age of 8.9 years, the volume of his testes began to increase, and other clinical signs of puberty appeared. He was diagnosed with precocious puberty through a gonadotropin-releasing hormone (GnRH) stimulation test. His bone age was still delayed at that time, but the lag behind chronological age had decreased by 1 year. The combination treatment of GnRH agonist (triptorelin) with GH began at the age of 10 years because of the rapid progression of puberty. He was treated with GH and GnRH agonist for 3 years; at the time of discontinuance of the GnRH agonist, his bone age was similar to the chronological age and his height SDS had increased to −0.88 SDS. He had no adverse events related with GnRH agonist treatment; moreover, his growth velocity did not decrease during treatment (Figure 1). As he was growing up, distinctive facial features suggesting KBG syndrome gradually became apparent. He showed macrodontia of the upper central incisors, a dysmorphic face including prominent and high nasal bridge, anteverted nostrils, hypertelorism, long philtrum, and brachydactyly of both hands (Figure 2A).

**FIGURE 3:**
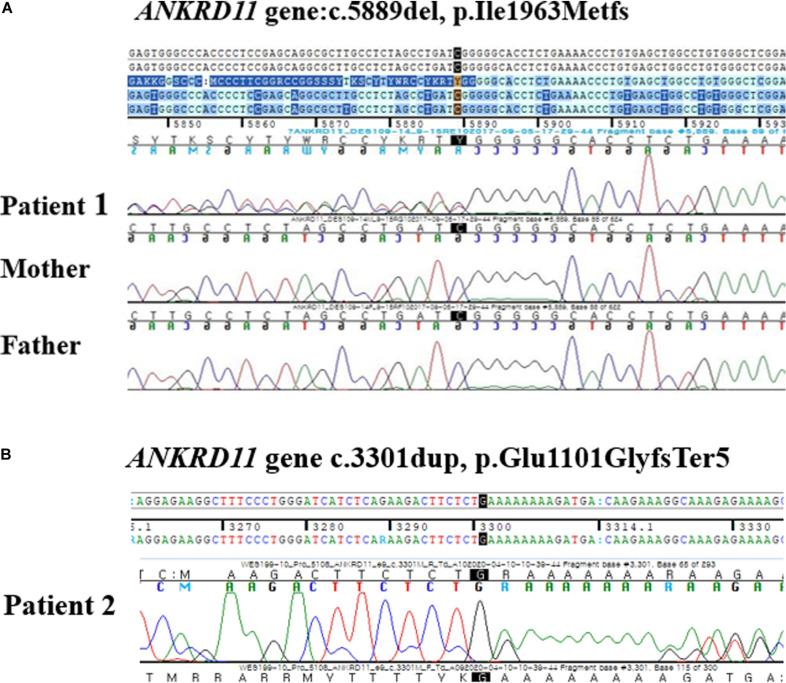
**(A)** Electropherograms of the patient 1 and his parents. A *de novo* heterozygous frameshift mutation at position 5889 (c.5889del) in the *ANKRD11* gene was found in the patient 1 and was not inherited from his parents. **(B)** Electropherograms of the patient 2. A heterozygous single base pair duplication at position 3301 (c.3301 dup) in the *ANKRD11* gene.

Targeted exome sequencing (TES) was performed and a novel frameshift variant was identified in ANKRD11, c.5889del, p. (Ile1963MetfsX9), which was confirmed with Sanger sequencing (Figure 3A). As the parental genetic tests revealed that the parents did not have this frameshift, the variation was identified as a *de novo* variant. This variant was categorized as pathogenic by the American College of Medical Genetics and Genomics (ACMG) guideline (PVS1, PS1, PS2, PM2) (Richards et al., 2015) and, to the best of our knowledge, has not been reported earlier. Regarding the neurologic and behavioral aspects, the patient had mild learning disability and had been taken methylphenidate for attention deficit hyperactivity disorder (ADHD) since childhood. However, he was able to participate in normal school life without any help.

### Patient 2

The patient was referred to our rare disease center at the age of 11 years to undergo evaluation for an undiagnosed neurodevelopmental disorder. He grew up in a child protective facility, so there was no information on his parents or birth history. He had seizures and progressive developmental delay from 3 months of age. He visited the genetics clinic of another hospital that performed tests for evaluation of developmental delay including chromosome analysis, skeletal survey, metabolic work up, brain magnetic resonance imaging (MRI), and electroencephalography (EEG). At that time, he was suspected with acrofacial dysostosis, catania type.

Despite taking an antiepileptic drug (oxcarbazepine 6.6 mg/kg/day), he had frequent focal epilepsy with secondary generalized convulsions. Brain MRI scans at the age of 3 months and 10 years showed no significant abnormal findings. EEG findings at the age of 3 months and 6.5 years suggested partial seizure disorder originating from the left occipital area. At the age of 7, his total intelligence quotient (TIQ), which was evaluated using the Korean version of the Wexler Intelligence (K-WISC III) and the Visual Motor Integrated Development Test, was about 26; he was considered to have a severe ID. He had behavioral problems such as autism-like features, anxiety, and stereotypical movements.

At the time of referral, he had normal height (149 cm, 0.77 SDS) and weight (42 kg, −0.09 SDS), and showed macrodontia of the permanent upper central incisors; dysmorphic face features such as triangular face, thick eye lashes and eye brows, prominent nasal bridge, long philtrum, and brachycephaly; and hand anomalies including brachydactyly and clinodactyly of the 5th finger (Figure 2B). His bone age was delayed compared to the chronological age according to the Greulich and Pyle atlas and there were no signs of puberty. He attended a special-education school with quite some assistance from his caregiver. He continued to take antiepileptic drugs (oxcarbazepine 33 mg/kg/day and levetiracetam 7 mg/day) and antipsychotic drugs (risperidone 3 mg/day and haloperidol 1.5 mg/day).

Whole exome sequencing (WES) was done, and a novel heterozygous variant, c3310dup, p. (Glu110GlyfsTer5) in ANKRD11 was identified (Figure 3B). In this variant, the amino acid glutamic acid is changed to glycine to result in a stop codon leading to nonsense-mediated decay, thereby causing haploinsufficiency of ANKRD11. This variant was categorized as likely pathogenic according to the ACMG guideline (PS1, PM2, PM4, PM5) (Richards et al., 2015).

### Mutational Analysis

A DNA sample was obtained from peripheral blood leukocytes by using a Chemagic Magnetic Separation Module I (Chemagic MSM I, BaesWeiler, Germany) extraction robot with DNA Blood 200 μL Kit. TruSight One (Illumina Inc., San Diego, CA) for patient 1 and SureSelect Human All Exon V5 (Agilent Technologies, Santa Clara, CA) for patient 2 were used for library preparation, and sequencing was done on the Illumina NextSeq500 platform (Illumina Inc., San Diego, CA) generating 2 × 150 bp paired-end reads. Alignment of sequence reads, indexing of the reference genome (hg19), and variant calling were performed with a pipeline based on the Genome Analysis Tool kit (GATK) Best Practices. Alignment was done with the BWA-mem (version 0.7.12), duplicated reads were marked with Picard (version 1.96)^1^ and local alignment, base quality recalibration, variant calling was performed with a GATK (version 3.5), and annotation was done with VEP88 (Variant Effect Predictor), dbNSFP v3.3.

## Discussion and Conclusion

There is currently no consensus regarding the clinical diagnostic criteria for KGB syndrome; however, several diagnostic criteria have been suggested (Skjei et al., 2007; Ockeloen et al., 2015; Low et al., 2016; Morel Swols et al., 2017). Postnatal short stature (defined as height below 3–10 percentile) is a major criterion, as 41–83% cases were described to show this characteristic in previous reports (Reynaert et al., 2015; Low et al., 2016; Morel Swols et al., 2017).

In 2015, Reynaert et al. reported first responses to GH treatment in KBG syndrome and suggested that GH would be a promising treatment for short stature in KBG syndrome, regardless of the presence of GH deficiency (Reynaert et al., 2015; Ge et al., 2020). Thereafter, some KBG syndrome patients with short stature have been treated with GH; preliminary evidence supports that GH treatment may be effective in enabling patients with KBG syndrome catch up to the normal growth rate (Goldenberg et al., 2016; Kang et al., 2019; Scarano et al., 2019).

Advanced puberty was also reported in a few patients with KBG syndrome (Goldenberg et al., 2016). Scarano et al. (2019) mentioned that early sign of puberty may influence short stature in KBG syndrome. It is not clear why the loss of function mutations of ANKRD11 gene causes short stature and advanced puberty. Our first patient was treated with GH and GnRH agonist due to GH deficiency combined with early and rapid progressive puberty. In prior reports, one male patient had been treated with GH and combined GnRH agonist, but the patient’s treatment started at 12.5 years of age and was stopped because of decreased growth velocity at 13.8 years (Reynaert et al., 2015). In another patient, treatment with GnRH agonist was interrupted after 2 months due to a local adverse effect (Scarano et al., 2019). In our case, the first pubertal signs presented when the patient was 8.9 years old, and the results of the GnRH test were consistent with a diagnosis of early puberty. With the accumulation of clinical data and the increase in the number of cases wherein KBG is diagnosed at a young age, the frequency of early puberty is likely to increase. Therefore, the careful monitoring of puberty-related signs in patients with KBG syndrome at pre and peripubertal ages is important. Further experience with GnRH agonist and/or GH treatment in KBG syndrome, including our case, will help establish guidelines about puberty and growth in patients with KBG syndrome.

ID is an important characteristic of KBG syndrome. The role of ANKRD11, which typically regulates transcription by binding chromatin-modifying enzymes such as histone deacetylases, in the development of the central nervous system, is not yet fully understood (Gallagher et al., 2015; Walz et al., 2015). Recently, Ka and Kim (2018) demonstrated a fundamental role of ANKRD11 in regulating pyramidal neuron migration and dendritic differentiation via the BDNF/TrkB signaling pathway in the developing mouse cerebral cortex. Haploinsufficiency of ANKRD11 due to microdeletion of chromosome 16q24.3 or loss of function mutation in the ANKRD11 gene results in the KBG syndrome phenotype, which includes neurodevelopmental problems. Alfieri et al. reported a low mean TIQ score (66.0 ± 16.2, represented as mean ± SD) in 17 patients with KBG syndrome (Alfieri et al., 2019), and van Dongen et al. (2017) showed similar mean TIQ distribution (66.5 ± 15.6 in 12 children, 62.5 ± 7.1 in 6 adults). As mentioned above, most patients have moderate to mild ID and more than half of the adults with KBG syndrome have jobs and are self-sufficient in ordinary life (Goldenberg et al., 2016; Low et al., 2016). Our second patient had severe ID (TIQ 26 at 7 years of age) with the lowest known TIQ in KBG syndrome up to date. Unfortunately, we did not have information about other environmental or perinatal factors related to ID, such as fetal alcohol exposure or perinatal asphyxia.

In addition to ID, some behavioral and psychopathologic problems such as ADHD, autism-like features, and impulsivity are major issues in patients with KBG syndrome (Novara et al., 2017). In recent study of 17 pediatric patients with KBG syndrome, there was a higher prevalence of obsessive-compulsive traits, tic disorders, depressive mood, and ADHD as compared with the general population; obsessive-compulsive traits, in particular, did not seem to be related to cognitive level (Alfieri et al., 2019). Similarly, another study also mentioned that ADHD-like behavioral problems associated with KBG syndrome could not be directly related or explained with a specific intelligence profile (van Dongen et al., 2017). In our cases, the first patient was treated for ADHD, and the second patient also had behavioral problems such as ASD, anxiety, and stereotypical movements.

As seen in our cases, some of the features of KBG syndrome including macrodontia and facial dysmorphism (triangular face, synophrys, thick eyebrow) have become more apparent over time. Facial dysmorphism in KBG syndrome are overlapping with that seen in other syndromes such as Cornelia de Lange syndrome or Kabuki syndrome. As it is not easy to suspect KBG syndrome based only on the characteristic gestalt, KBG syndrome is considered vulnerable to underdiagnosis. Recently, the number of cases of KBG syndrome being diagnosed before 6 years of age through a next-generation sequencing (NGS) panel or WES has increased. In these cases, the clinical symptoms of KBG syndrome such as macrodontia and short stature were not clear. Developmental delay, especially speech delay and congenital malformations such as congenital heart defect or urogenital anomalies, hearing loss, and feeding difficulties are the main clinical manifestations in younger patients (Goldenberg et al., 2016; Low et al., 2016; Gnazzo et al., 2020). Low et al. recommended including the ANKRD11 gene in the NGS panel for developmental delay (Low et al., 2016). Early diagnosis through NGS or TES can enable clinicians to monitor possible problems, such as behavioral disorders, short stature, and hearing impairment. Genetic counseling is also of vital importance in the management of this condition. Early interventions may improve the patient’s quality of life.

In conclusion, we report two novel mutations of the ANKRD11 gene with different phenotypes of KBG syndrome. GH treatment and combined GnRH agonist treatment are effective in a child with KBG syndrome with short stature and early puberty. Moreover, our case presented with severe ID, seizures, and psychopathologic problems without short stature despite KBG syndrome being generally characterized by mild to moderate ID. Considering the various phenotypes of KBG syndrome, a NGS panel including the ANKRD11 gene for developmental delay with/without short stature may be helpful to identify patients with undiagnosed KBG syndrome.

## Data Availability Statement

The datasets for this article are not publicly available due to concerns regarding participant/patient anonymity. Requests to access the datasets should be directed to the corresponding author.

## Ethics Statement

Ethics board approval and consent was obtained for this work from the Institutional Review Board at the Inha University Hospital, Incheon, Korea (2020-03-039). The patients’ parent or the parental agent provided written informed consent. Written informed consent was obtained from the patient’s parent or the parental agent for publication of this case report and any accompanying images.

## Author Contributions

SJK analyzed and interpreted the data, prepared and corrected the manuscript. YSK and JEL designed the study. AY, J-SL, and DGK involved in the revision of this manuscript. All authors read and approved the final manuscript.

## Conflict of Interest

The authors declare that the research was conducted in the absence of any commercial or financial relationships that could be construed as a potential conflict of interest.

## Footnotes

1 http://picard.sourceforge.net
